# Donor-Derived West Nile Virus Infection in Kidney Transplant Recipients, France, 2025

**DOI:** 10.3201/eid3202.251569

**Published:** 2026-02

**Authors:** Aurélie Truffot, Anne-Sophie Montcuquet, Gilda Grard, Laura Pezzi, Raphaelle Klitting, Gabriel Clavel, Sylvain Trincal, Paolo Malvezzi, Lionel Rostaing, Lara Meyer, Stéphanie Dieterle, Sophie Lucas Samuel, Vincent Foulongne, Laura Le Fleur, Patricia Pavese, Gaël Pradel, Florian Franke, Syria Laperche, Julien Lupo, Anne Signori-Schmuck, Raphaële Germi, Patrice Morand

**Affiliations:** Université Grenoble Alpes, CNRS, CEA, IRIG IBS, Laboratoire de virologie, Grenoble, France (A. Truffot, J. Lupo, A. Signori-Schmuck, R. Germi, P. Morand); Université Grenoble Alpes, CHU Grenoble Alpes, Service de néphrologie, Grenoble (A.-S. Montcuquet, G. Clavel, S. Trincal, P. Malvezzi, L. Rostaing); Centre National de Référence des Arbovirus, Inserm-IRBA, Marseille, France (G. Grard, L. Pezzi, R. Klitting); Unité des Virus Émergents, Aix-Marseille Université, Universita di Corsica, IRD, Inserm, Marseille (G. Grard, L. Pezzi, R. Klitting); Service de Néphrologie, Montpellier, France (L. Meyer); Direction Générale Médicale et Scientifique, Agence de la Biomédecine, Saint-Denis, France (S. Dieterle, S.L. Samuel); Centre Hospitalier Universitaire de Montpellier, Montpellier (V. Foulongne, L. Le Fleur); Grenoble-Alpes University Hospital, La Tronche, France (P. Pavese); Centre Hospitalier d’Avignon Henri Duffaut, Avignon, France (G. Pradel); Santé Publique France, Marseille (F. Franke); Etablissement Français du Sang, Saint-Denis (S. Laperche)

**Keywords:** West Nile virus, vector-borne infections, viruses, solid organ transplantation, arboviruses, France

## Abstract

We report 2 cases of donor-derived West Nile virus infection in kidney transplant recipients in France. Both recipients had mild disease develop and recovered without sequelae. A more proactive screening strategy in France, particularly during periods of highest risk for West Nile virus circulation, would help reduce risk for donor-derived infections.

The clinical manifestation of West Nile virus (WNV) infections is a wide spectrum, ranging from asymptomatic (≈80% of cases) to mild febrile illness (fever, rash) in ≈20% of cases; severe neuroinvasive disease develops in a small proportion of patients (*1*). Immunocompromised patients, such as solid organ transplant (SOT) recipients, are at higher risk for complicated neuroinvasive forms of WNV with unfavorable outcomes (*2*–*4*).

In France, the High Council for Public Health updated its recommendations in 2020 for transfusion and transplant safety in relation to WNV circulation (https://www.hcsp.fr/Explore.cgi/AvisRapports). According to those guidelines, individual screening of human-derived biologic products relies at minimum on WNV genome detection, combined with IgM testing for organ donors. Annually, in mainland France, screening measures are implemented at the department level after the first human autochthonous case, regardless of history of WNV circulation. Since 2024, during the period of enhanced surveillance of arboviruses (May–November), a decision-making support unit convenes weekly to discuss adapting screening strategies in light of reported detections (https://cartosan.fr/rs). In this article, we describe 2 cases of donor-derived West Nile virus (DD-WNV) infection in kidney transplant recipients from a donor in France and discuss the current strategy for WNV screening of human-derived biologic products.

## The Study

In August 2025, a 38-year-old woman was admitted to Grenoble University Hospital (Grenoble, France) with acute kidney failure, fever, headaches, and diarrhea occurring 3 weeks after left kidney transplantation. Extensive microbiological investigations detected WNV genome in multiple specimens and WNV IgM in serum collected 14 days after symptom onset, whereas serum collected just before kidney transplantation was negative by quantitative reverse transcription PCR (RT-PCR) and serology (WNV IgM and IgG) (Appendix Figure). A graft biopsy showed mild capillaritis affecting both glomeruli and tubules, along with granular tubular casts consistent with infection; WNV genome was also detected in the kidney biopsy specimen. Fever and headache resolved within a few days, and no neurologic signs were noted. Fifteen days later, kidney function remained impaired but stable, and she was discharged (Appendix Figure). WNV quantitative RT-PCR of urine still showed positive result 2.5 months after the graft. During the transplant, the recipient received twice-packed red blood cells that retrospectively tested negative for WNV by quantitative RT-PCR.

The right kidney recipient, a 61-year-old man, was admitted to Montpellier University Hospital (Montpellier, France) 20 days after transplantation, in August 2025, with isolated fever without neurologic symptoms. Virologic tests were negative except for WNV-positive quantitative RT-PCR results in whole blood, urine, saliva, and serology (WNV IgM and IgG) 24 days after transplant. Fever spontaneously resolved within 5 days, and the patient was discharged with favorable outcome (Appendix Figure).

**Figure 1:**
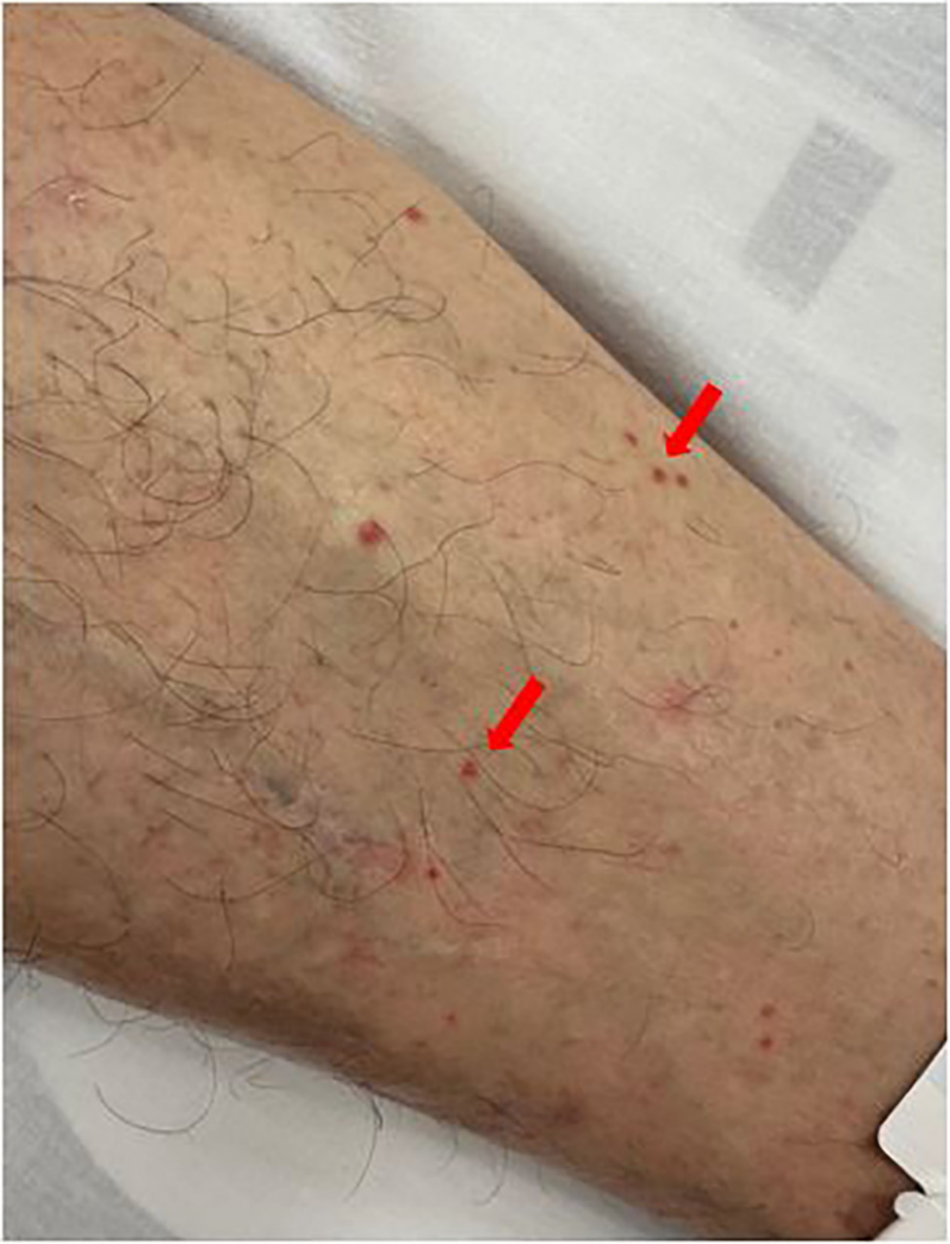
Diffuse skin lesions on donor’s leg in case of donor-derived West Nile virus infection in kidney transplant recipients, France, 2025. Lesions were suggestive of eruptive pseudo-angiomatosis associated with West Nile virus infection.

Because DD-WNV infection was suspected, the cases were reported to the French Biomedicine Agency, the national authority responsible for regulating and supervising organ, tissue, and cell donation and transplantation. The organ donor was a man in his 50s who died mid-July 2025 from a cerebral hemorrhage caused by hypertensive crisis. On the day of death, he demonstrated no suggestive clinical signs except for lesions on his abdomen and lower limbs suggestive of eruptive pseudo-angiomatosis (Figure 1). Only the kidneys were retrieved and grafted. Donor specimens collected just before organ donation were retrospectively tested; WNV genome was identified in both serum and plasma, whereas IgM and IgG were negative, confirming ongoing WNV infection (Appendix Figure).

**Figure 2:**
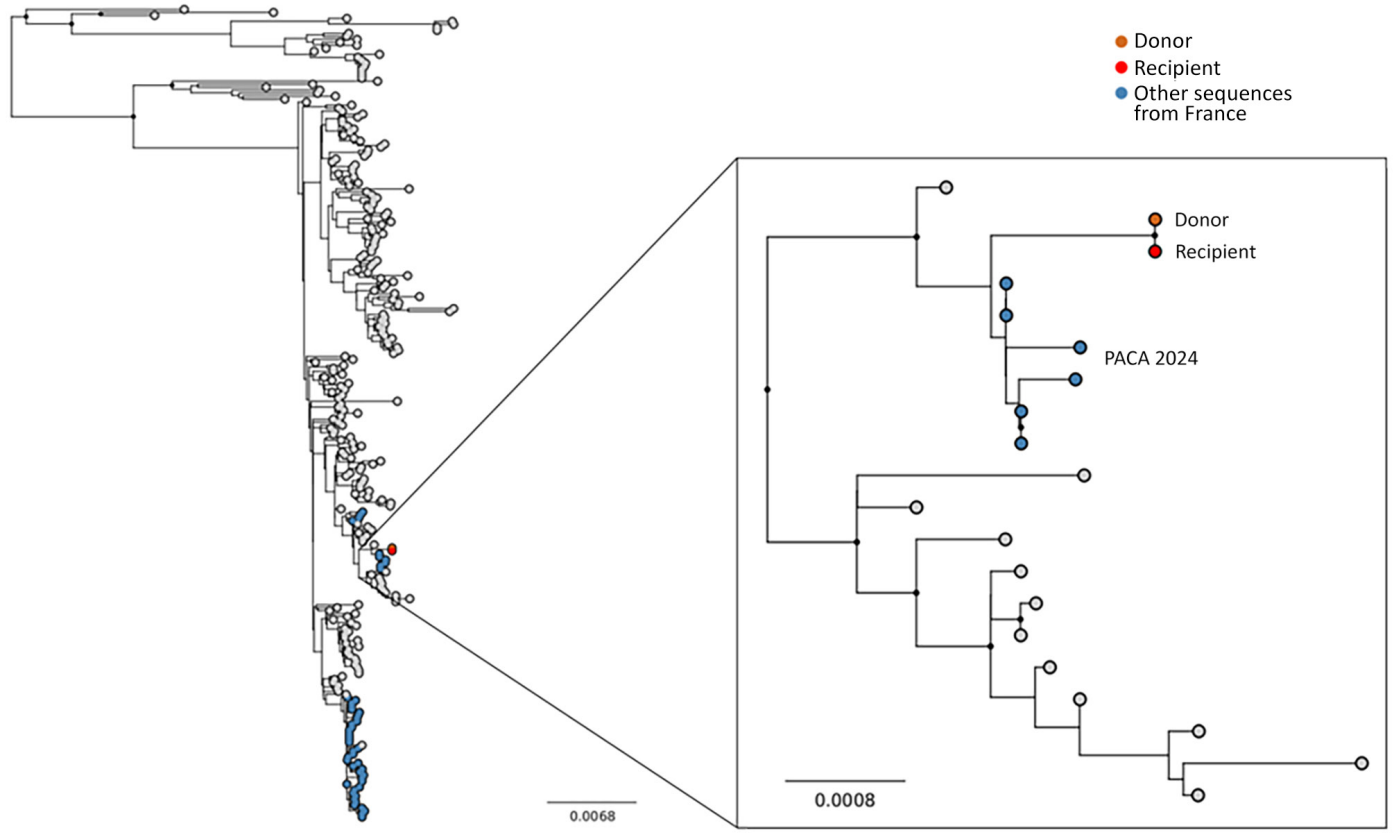
Phylogeny of West Nile virus lineage 2 highlighting sequences from the infected donor and recipient in case of donor-derived West Nile virus infection in kidney transplant recipients, France, 2025. Maximum-likelihood phylogeny was reconstructed with IQ-TREE version 1.6.12 (https://iqtree.github.io/release/v1.6.12). The best-fit nucleotide substitution model was selected using ModelFinder (https://iqtree.github.io/ModelFinder), and branch support was evaluated with ultrafast bootstrap approximation using 1,000 replicates. We used 226 sequences representative of the phylogenetic diversity within lineage 2 (subsampled from a comprehensive set of public sequences from GenBank accessed via Pathoplexus, https://doi.org/10.62599/PP_SS_67.1), including those from the donor and recipient produced in this study, as well as other sequences from France, including those from Provence-Alpes Côte d’Azur (PACA) region. The length of the branches is proportional to the degree of divergence.

DD-WNV was confirmed by next-generation sequencing of complete WNV genomes identified in the donor’s plasma and in left kidney recipient’s urine (GenBank accession nos. OZ313260.1, OZ313201.1) (*5*). Unfortunately, the viral load identified in the right kidney recipient was too low to enable virus genome sequencing. Virus genomes obtained from the donor and the recipient were 100% identical, confirming a direct link between infections. Further phylogenetic inference using a maximum-likelihood approach showed that the virus strain responsible for the initial infection belonged to WNV lineage 2 and was related to strains sampled in the Provence-Alpes-Côte d’Azur region in 2024, aligning with the donor’s region of residence rather than with that of the kidney recipient (Auvergne-Rhône-Alpes) (Figure 2).

For the left kidney recipient, WNV genome was detected by RT-PCR using the ELITe MBG kit (ELITech Group, https://www.elitechgroup.com), and WNV IgM and IgG were detected using the Vircell VIRCLIA monotest (https://www.vircell.com). For the right kidney recipient, WNV genome was detected by RT-PCR Altona Real Star kit (https://altona-diagnostics.com), and serologic testing was performed with the EUROIMMUN ELISA (https://www.euroimmun.com). The results were confirmed by the National Reference Center using an in-house WNV quantitative RT-PCR on the Panther Fusion system (Hologic, https://www.hologic.com) and the EUROIMMUN ELISA for WNV serology.

The first cases of DD-WNV were reported in the United States in 2002, involving 4 SOT recipients from a common donor (*4*). Through 2017, WNV was estimated to account for >6% of DD infections occurring in kidney recipients (*6*). Review of previous cases indicates that 85% of SOT recipients from WNV-infected donors acquire the infection; 70% develop neuroinvasive disease, and the mortality rate is 26% (*4*). In this instance, both kidney recipients developed a mild infection and had favorable outcomes and no sequelae.

Laboratory investigations confirmed WNV infection in both recipients, and genomic and phylogenetic analysis demonstrated DD-WNV. Of note, genome testing of whole blood was more sensitive than plasma testing, consistent with previous findings (*7*–*10*). The RT-PCR–positive result in saliva suggests this type of specimen can be useful for WNV genome detection, although the diagnostic value of saliva warrants further investigation (*11*).

The donor had not recently traveled to known WNV risk areas and resided in the Vaucluse department, where no WNV activity had been reported at the time of organ donation. The first department identified as at risk in 2025 was Var, which was added to the list of at-risk areas in July (after transplant) (*12*). This donor was retrospectively classified as the first autochthonous case of WNV infection in Vaucluse department, which was added to the list in August 2025 (*13*). He had not received recent blood transfusions, and the lesions observed at hospital admission have been described in association with various viral infections, including WNV (*14*). Vectorborne transmission is the most likely acquisition scenario. Equine cases of WNV were confirmed in July 2025 <30 km from the donor’s residence, providing additional evidence of active WNV circulation at the time of death (https://www.plateforme-esa.fr/fr/bulletin-hebdomadaire-de-veille-sanitaire-internationale-du-05-08-2025). 

## Conclusions

This case underscores the limitations of WNV surveillance in France, particularly regarding the timing of screening and the criteria used to define areas requiring mandatory screening. The current reactive approach, in which screening of human-derived biologic products is mandatory only after the first human autochthonous case is identified, is limited by the high proportion of asymptomatic WNV infections in humans (*1*). In areas with recurrent WNV circulation and favorable conditions, a proactive and systematic screening of human-derived biologic products during the vector activity season would likely be more appropriate, provided it is financially feasible and that laboratories have adequate diagnostic capacity. This approach was successfully implemented during the 2024 Summer Olympics, when the public transfusion service in France introduced WNV nucleic acid testing for blood donations in areas with documented previous viral circulation, and the first PCR-positive blood donation was identified 3 weeks before the first human symptomatic case (*15*). Conversely, in regions with no evidence of WNV circulation, a reactive approach seems sufficient to prevent transmission through transfusion and transplants. Both proactive and reactive screening strategies would greatly benefit from entomological and animal surveillance, which might detect WNV circulation before the onset of human cases (*5*). Integrating such early alerts into the decision-making process for initiating WNV donor screening would further help reduce the risk for WNV transmission.

Appendix Additional information about donor-derived West Nile virus infection in kidney transplant recipients, France, 2025

## References

[1] Sejvar JJ. West Nile virus infection. Microbiol Spectr. 2016;4:4. 27337465 10.1128/microbiolspec.EI10-0021-2016

[2] Abbas A, Qiu F, Sikyta A, Fey PD, Florescu DF. Neuroinvasive West Nile virus infections after solid organ transplantation: Single center experience and systematic review. Transpl Infect Dis. 2022;24:e13929. 10.1111/tid.13929 35980220 PMC10078393

[3] Kumar D, Prasad GVR, Zaltzman J, Levy GA, Humar A. Community-acquired West Nile virus infection in solid-organ transplant recipients. Transplantation. 2004;77:399–402. 10.1097/01.TP.0000101435.91619.31 14966414

[4] Soto RA, McDonald E, Annambhotla P, Velez JO, Laven J, Panella AJ, et al. West Nile virus transmission by solid organ transplantation and considerations for organ donor screening practices, United States. Emerg Infect Dis. 2022;28:403–6. 10.3201/eid2802.211697 34843660 PMC8798677

[5] Bigeard C, Pezzi L, Klitting R, Ayhan N, L’Ambert G, Gomez N, et al. Molecular Xenomonitoring (MX) allows real-time surveillance of West Nile and Usutu virus in mosquito populations. PLoS Negl Trop Dis. 2024;18:e0012754. 10.1371/journal.pntd.0012754 39724146 PMC11709297

[6] Shingde R, Habachou LI, Calisa V, Craig JC, Tong A, Chen SC, et al. Unexpected donor-derived infectious transmissions by kidney transplantation: A systematic review. Transpl Infect Dis. 2018;20:e12851. 10.1111/tid.12851 29508947

[7] Barzon L, Pacenti M, Franchin E, Squarzon L, Sinigaglia A, Ulbert S, et al. Isolation of West Nile virus from urine samples of patients with acute infection. J Clin Microbiol. 2014;52:3411–3. 10.1128/JCM.01328-14 24951801 PMC4313171

[8] Lustig Y, Mannasse B, Koren R, Katz-Likvornik S, Hindiyeh M, Mandelboim M, et al. Superiority of West Nile virus RNA detection in whole blood for diagnosis of acute infection. J Clin Microbiol. 2016;54:2294–7. 10.1128/JCM.01283-16 27335150 PMC5005505

[9] Rios M, Daniel S, Chancey C, Hewlett IK, Stramer SL. West Nile virus adheres to human red blood cells in whole blood. Clin Infect Dis. 2007;45:181–6. 10.1086/518850 17578776

[10] Lanteri MC, Lee TH, Wen L, Kaidarova Z, Bravo MD, Kiely NE, et al. West Nile virus nucleic acid persistence in whole blood months after clearance in plasma: implication for transfusion and transplantation safety. Transfusion. 2014;54:3232–41. 10.1111/trf.12764 24965017 PMC4268370

[11] Gorchakov R, Gulas-Wroblewski BE, Ronca SE, Ruff JC, Nolan MS, Berry R, et al. Optimizing PCR detection of West Nile virus from body fluid specimens to delineate natural history in an infected human cohort. Int J Mol Sci. 2019;20:1934. 10.3390/ijms20081934 31010160 PMC6514913

[12] Agence de la Biomédecine. Update to the list of countries and territories identified as being at risk of West Nile virus (WNV) transmission–2025 season–addition of the Var department [in French] [cited 2025 Aug 14]. https://www.santepubliquefrance.fr/maladies-et-traumatismes/maladies-a-transmission-vectorielle/chikungunya/documents/bulletin-national/chikungunya-dengue-zika-et-west-nile-en-france-hexagonale.-bulletin-de-la-surveillance-renforcee-du-27-aout-2025

[13] Agence de la Biomédecine. Update to the list of countries and territories identified as being at risk of West Nile virus (WNV) transmission—2025 season—addition of the Vaucluse department [in French] [cited 2025 Aug 14]. https://www.hcsp.fr/explore.cgi/avisrapportsdomaine?clefr=1437

[14] Pacenti M, Sinigaglia A, Franchin E, Pagni S, Lavezzo E, Montarsi F, et al. Human West Nile virus lineage 2 infection: epidemiological, clinical, and virological findings. Viruses. 2020;12:458. 10.3390/v12040458 32325716 PMC7232435

[15] Grard G, Franke F, Laperche S, Cochet A, Paty MC, Gonzalez G, et al. Blood donation screening and West Nile virus surveillance strategy in France. JAMA Netw Open. 2025;8:e2524494. 10.1001/jamanetworkopen.2025.24494 40742595 PMC12314714

