# Supplementary material for: Donor-Derived West Nile Virus Infection in Kidney Transplant Recipients, France, 2025
Source: Emerg Infect Dis. 2026 Feb;32(2):281–4. doi: 10.3201/eid3202.251569 (PMC12928219; doi:10.3201/eid3202.251569)
Supplement: Appendix — Additional information about donor-derived West Nile virus infection in kidney transplant recipients, France, 2025 [file 25-1569-Techapp-s1.pdf]

Article DOI: https://doi.org/10.3201/eid3202.251569

EID cannot ensure accessibility for supplementary materials supplied by authors. Readers who have difficulty accessing supplementary content should contact the authors for assistance.

Donor-Derived West Nile Virus Infection in Kidney Transplant Recipients, France, 2025

Appendix

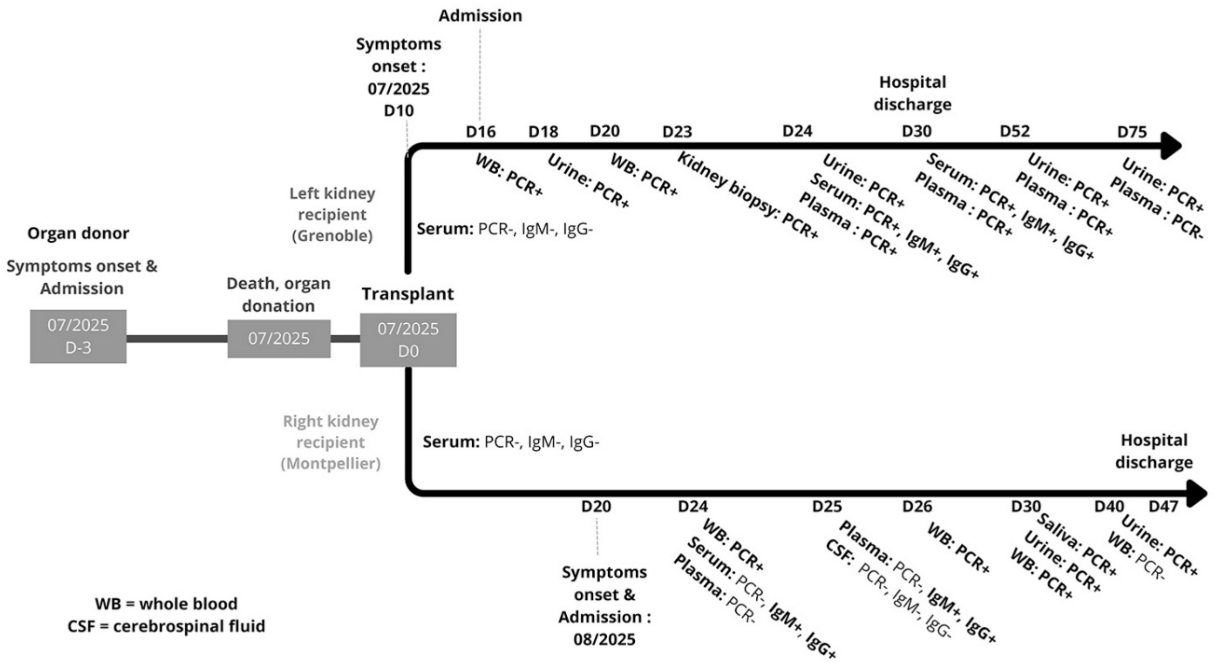

Appendix Figure. Timeline showing the results of all biologic tests performed on donor and 2 recipients in case of donor-derived West Nile virus infection in kidney recipients, France, 2025. Combined results of analyses from the National Reference Center (in-house WNV RT-qPCR test on Panther Fusion; EUROIMMUN ELISA test), the Grenoble laboratory (RT-PCR ELITe MGB kit® - Vircell VIRCLIA monotests for serology) and the Montpellier laboratory (RT-PCR Altona Real Star kit® - EUROIMMUN ELISA test).
